# Structural basis for Sfm1 functioning as a protein arginine methyltransferase

**DOI:** 10.1038/celldisc.2015.37

**Published:** 2015-12-29

**Authors:** Fengjuan Lv, Tianlong Zhang, Zhen Zhou, Shuaixin Gao, Catherine CL Wong, Jin-Qiu Zhou, Jianping Ding

**Affiliations:** 1 National Center for Protein Science Shanghai, State Key Laboratory of Molecular Biology, Institute of Biochemistry and Cell Biology, Shanghai Institutes for Biological Sciences, Chinese Academy of Sciences, Shanghai, China

**Keywords:** Arginine methylation, PRMT, ribosomal protein S3, ribosome assembly, SPOUT

## Abstract

SPOUT proteins constitute one class of methyltransferases, which so far are found to exert activity mainly towards RNAs. Previously, yeast Sfm1 was predicted to contain a SPOUT domain but can methylate ribosomal protein S3. Here we report the crystal structure of Sfm1, which comprises of a typical SPOUT domain and a small C-terminal domain. The active site is similar to that of protein arginine methyltransferases but different from that of RNA methyltransferases. In addition, Sfm1 exhibits a negatively charged surface surrounding the active site unsuitable for RNA binding. Our biochemical data show that Sfm1 exists as a monomer and has high activity towards ribosomal protein S3 but no activity towards RNA. It can specifically catalyze the methylation of Arg146 of S3 and the C-terminal domain is critical for substrate binding and activity. These results together provide the structural basis for Sfm1 functioning as a PRMT for ribosomal protein S3.

## Introduction

Methylation of biological molecules, such as proteins, nucleic acids, lipids and small molecules, is one of the most common modifications, and has various important roles in many cellular processes, including heterochromatin formation, transcription, RNA processing, DNA repairing, protein metabolism and cellular signaling [1, 2]. A large family of enzymes called methyltransferases (MTases) catalyze the addition of a methyl group to a nucleophilic acceptor mainly using *S*-adenosyl-methionine as the cofactor [1]. MTases exist ubiquitously in all organisms [3], and most of the enzymes belong to the seven-beta-strand, SET and SPOUT MTases [4]. The seven-beta-strand MTases, which constitute the largest group of MTases, can catalyze the methylation of a wide range of substrates, including proteins, nucleic acids, lipids and small molecules [5]. The SET MTases are responsible for most of lysine methylation of histones [6], and additionally can catalyze the methylation of some non-histone proteins such as transcription factors and ribosomal proteins [7]. So far, the SPOUT MTases are found to exert activity only towards RNAs [8].

In eukaryotes, methylation modification of proteins can take place at the side chains of several amino acids such as arginine, lysine, histidine, glutamate and cysteine [7]. Arginine methylation is one of the most common protein methylation modifications, and the methylation can occur at three guanidino nitrogen atoms of the side chain, which is catalyzed by a group of structurally conserved enzymes called protein arginine MTases [9, 10]. In mammals, there are nine PRMTs identified so far, named as PRMT1-9. PRMT1, PRMT2, PRMT3, PRMT4, PRMT6 and PRMT8 belong to type I PRMTs that catalyze the MMA (ω-*N*^G^-monomethylarginine) and aDMA (ω-*N*^G^, *N*^G^-asymmetric dimethylarginine) modifications; PRMT5 and PRMT9 are type II PRMTs that catalyze the MMA and sDMA (ω-*N*^G^, *N*’^G^-symmetric dimethylarginine) modifications; and PRMT7 is a type III PRMT that catalyzes only the MMA modification [9, 11, 12]. In *Saccharomyces cerevisiae,* there are four PRMTs identified so far, namely Rmt1, Hsl7, Rmt2 and Sfm1 [10]. Rmt1 is a type I PRMT [13]; Hsl7 is a type II PRMT [14]; and Rmt2 is a type IV PRMT that can specifically catalyze the δ-MMA (δ-*N*-monomethylarginine) modification [15, 16]. Structural studies have shown that all of these PRMTs belong to the seven-beta-strand class of MTases [17–20]. Intriguingly, Sfm1 was found to be able to catalyze ω-monomethylation of Arg146 of yeast ribosomal protein S3 [21] but was predicted to contain a SPOUT domain [8].

Ribosomal protein S3 is an essential component of the small subunit of eukaryotic and prokaryotic ribosome. In addition, it has important roles in many cellular processes including DNA repairing, gene regulation and immune response [22–24]. Human S3 could be methylated at Arg64, Arg65 and Arg67 by PRMT1, and the methylations have a critical role in its import into the nucleolus and in ribosome assembly [25]. Human S3 shares about 66% sequence identity with yeast S3 and also contains a conserved Arg146. Whether Arg146 of human S3 could be methylated is unknown and what is the functional role of Arg146 methylation of S3 is also unclear.

In this work, we carried out the structural and functional studies of Sfm1. We show that indeed Sfm1 consists of a typical SPOUT domain at the N-terminus flanked by a small C-terminal domain (CTD). Sfm1 exists as a monomer and exhibits a negatively charged surface surrounding the active site unsuitable for RNA binding. The active site is also similar to that of PRMTs but different from that of RNA MTases. Consistently, Sfm1 has no activity towards RNAs but can specifically catalyze the Arg146 methylation of yeast and human S3, and the CTD is critical to the substrate binding and the activity. Moreover, our *in vivo* functional data suggest that the Arg146 methylation has an important role in the import of human S3 into the nucleolus. These results together provide the structural basis for the SPOUT protein Sfm1 functioning as a PRMT for the Arg146 methylation of ribosomal protein S3.

## Results

### Overall structure of Sfm1

The crystal structure of a C-terminal truncated Sfm1 (Sfm1ΔC: residues 1−204) in apo form was solved by the single-wavelength anomalous dispersion method at 2.0 Å resolution and refined at 1.9 Å resolution, and the crystal structure of the full-length Sfm1 (residues 1−213) in complex with *S*-adenosyl-homocysteine (SAH) was determined by the molecular replacement method at 2.5 Å resolution (Table 1). These are two Sfm1 molecules in an asymmetric unit (ASU) in both the apo and SAH-bound Sfm1 structures, which assume almost identical overall structure with a root-mean-square deviation of 1.0 and 0.3 Å, respectively. In the apo Sfm1 structure, all residues of one Sfm1 (molecule A) are well defined; however, a significant portion of the other Sfm1 (molecule B) are undefined owing to poor electron density (residues 79−99, 106−136, 152−162, 185 and 187−189). In both molecules, the C-terminal his-tag (5 residues) and the linker (2 residues) are defined, which are involved in interactions with an adjacent molecule. In the SAH-bound Sfm1 structure, both molecules are well defined except for residues 1 and 205−213 of molecule A and residues 1, 172−175 and 205−213 of molecule B, and the C-terminal his-tag and the linker are disordered in both molecules.

**Figure 1:**
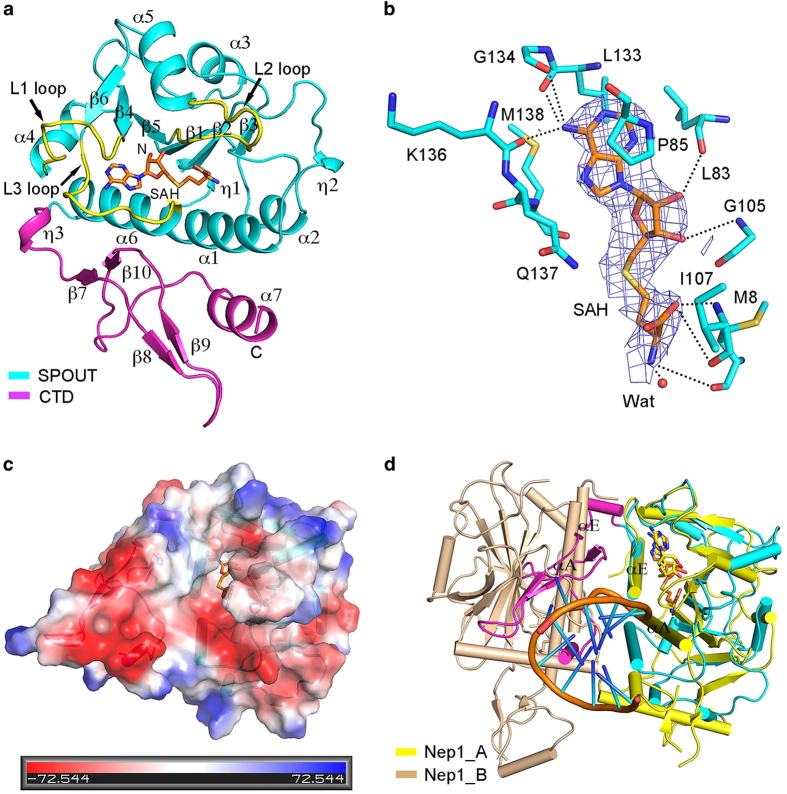
Structure of the SAH-bound Sfm1. (**a**) Overall structure of the SAH-bound Sfm1. The SPOUT domain and the CTD are colored in cyan and magenta, respectively, and the secondary structure elements are marked. The L1, L2 and L3 loops involved in the cofactor binding are highlighted in yellow. SAH is shown with a stick mode and colored in orange. (**b**) Interactions of SAH with the surrounding residues. The hydrogen-bonding interactions are indicated with black dashed lines. The simulated annealing composite 2Fo-Fc omit map (blue) for SAH is shown with the blue grids (contoured at 1.0σ). (**c**) Electrostatic potential surface of the SAH-bound Sfm1. The surface charge distribution is displayed as blue for positive, red for negative and white for neutral. The structure of Sfm1 is shown with a ribbon model and SAH is shown with a stick model. (**d**) Superposition of the SAH-bound Sfm1 with the Nep1-RNA complex. The SPOUT domains of the two proteins can be superimposed very well with an root-mean-square deviation of 3.2 Å for 117 Cα atoms. Two monomers of the Nep1 homodimer are colored in yellow and wheat, respectively. For clarity, only one RNA molecule in the dimeric Nep1-RNA complex is shown with a ribbon model and colored in orange. The two α-helices (αA and αE) of Nep1 at the dimer interface are indicated.

Sfm1 consists of two domains: an N-terminal domain and a small CTD. The N-terminal domain (residues 1−154) is composed of a central twisted six-stranded parallel β-sheet (β1−β6) sandwiched by four α-helices on one side (α1, α2, α4 and α6) and two α-helices on the other side (α3 and α5). The CTD (residues 155−213, CTD) is composed of four short β-strands (β7−β10) and a short α-helix (α7, which folds along one side of the N-terminal domain (Figure 1a and Supplementary Figure S1a). Structural similarity search using the DALI server [26] reveals that the N-terminal domain of Sfm1 shares the highest structural similarities with the SPOUT domains, including *Schizosaccharomyces pombe* Trm10 [27], *Escherichia coli* TrmL [28], *Saccharomyces cerevisiae* Nep1 [29], and *Haemophilus influenza* TrmD [30] (Supplementary Table S1). This is in agreement with the previous prediction that Sfm1 contains a SPOUT domain at the N terminus [8]. In the SPOUT domain of Sfm1, the N-terminal region (β1−β3 and α1−α3) forms the α/β fold and the C-terminal region (β4−β6, α4−α6 and the three connecting loops) forms the deep trefoil knot [8, 31, 32] (Figure 1a).

### Structure of the cofactor-binding site

In the SAH-bound Sfm1 structure, SAH is clearly defined in the electron density map (Figure 1b). SAH binds to a pocket formed by the three connecting loops of the trefoil knot (residues 83−92, 105−115 and 131−140, referred as L1, L2 and L3 loops, respectively), and assumes a bent conformation (Figure 1a), similar to that in the structures of other SPOUT MTases [27–30]. The adenine moiety of SAH has largely hydrophobic interactions with Pro85 of the L1 loop and Leu133 and Met138 of the L3 loop, and additionally the N6 group of the adenine moiety forms three hydrogen bonds with the main-chain carbonyl groups of Leu133, Gly134 and Lys136 of the L3 loop (Figure 1b). The 2'-OH and 3'-OH groups of the ribose moiety form a hydrogen bond with the main-chain carbonyl group of Leu83 of the L1 loop and the main-chain amine group of Gly105 of the L2 loop, respectively. The homocysteine moiety interacts with the main-chain amine and carbonyl groups of Ile107 of the L2 loop via its carboxyl group and interacts with the main-chain carbonyl group of Met8 of the β1-α1 loop and a water molecule via its amine group. Sequence alignment shows that the key residues involved in SAH binding are highly conserved in Sfm1 from different species (Supplementary Figure S1b).

Structural comparison shows that the apo and the SAH-bound Sfm1 assume very similar overall structure (root-mean-square deviation of 1.2 Å for 202 Cα atoms); however, the SAH binding induces some notable conformational changes at the active site (Supplementary Figure S1c). Particularly, on the cofactor binding, the β5-α5 or L2 loop, the following α5 helix, and the β1-α1 loop move towards SAH by about 3.0 Å. These conformational changes lead to formation of a more compact active site and thus several residues of the two loops are either involved in interactions with SAH or in appropriate positions to interact with the substrate.

### Sfm1 exists as a monomer in structure and solution

Up to date, most of the SPOUT MTases exist and function as homodimers and the dimerization is essential for substrate binding and activity [8]. The dimer interface is mainly mediated by two parallel α-helices (corresponding to α1 and α6 in Sfm1) of each monomer, which are arranged in either ‘perpendicular’ or ‘antiparallel’ manner to form a four-helix bundle [8]. TrmL is a typical SPOUT MTase with the ‘perpendicular’ [28] manner and TrmD with the ‘antiparallel’ manner [30] (Supplementary Figures S2a and b). Most recently, the tRNA MTase Trm10 was found to exist and function as a monomer, which contains an extra C-terminal α-helix blocking the dimer interface in the dimeric TrmH (in ‘perpendicular’ manner) and TrmD (in ‘antiparallel’ manner) [27]. Interestingly, Sfm1 shares the highest structural similarity with Trm10 and also contains an extra CTD.

Although there are two Sfm1 molecules per ASU in both the apo and SAH-bound Sfm1 structures, the intermolecular interfaces in these two structures are very different (Supplementary Figure S3), which are also different from those in the dimeric TrmL and TrmD (Supplementary Figure S2). In the apo Sfm1 structure, the two molecules in the ASU are arranged in a non-symmetric manner (related by a rotation of 145°) and the intermolecular interface involves mainly the C-terminal his-tag, α7 and β9 of the CTD, and the β1-α1 loop of molecule A, and the C-terminal his-tag, the linker, and α7 and β9 of the CTD of molecule B (Supplementary Figure S3a). In the SAH-bound Sfm1 structure, the two molecules in the ASU are related by a pseudo two-fold symmetry (about 180°) and the intermolecular interface involves mainly α1, α6 and η1 (or the equivalent loop) of the SPOUT domain in both molecules (Supplementary Figure S3b). Superposition of the Sfm1 monomer with the TrmL and TrmD homodimers shows that similar to Trm10, the CTD of Sfm1 would have steric clashes with the two α-helices of the other monomer at the dimer interface in TrmL and TrmD, explaining why Sfm1 cannot form a homodimer in either the ‘perpendicular’ or ‘antiparallel’ mode (Supplementary Figure S2c and d). In addition, the intermolecular interface (between the SPOUT domains) buries about 730 and 1431 Å^2^ solvent accessible surface in the apo and SAH-bound Sfm1 structures, respectively, which are much smaller than the dimer interfaces (between the SPOUT domains) in the dimeric SPOUT MTases (2 670 Å^2^ in TrmL, 2 732 Å^2^ in Nep1 and 2 427 Å^2^ in TrmD) as calculated using the PISA server [33]. Moreover, our analytical gel filtration chromatography and dynamic light scattering analyses show that Sfm1 exists as a monomer in solution, and the C-terminal truncation, different fusion tag locations, and binding with or without SAH have no effects on its oligomerization state (Supplementary Figure S4a and c). These data together indicate that similar to Trm10, Sfm1 exists as a monomer in both structure and solution.

### Sfm1 does function as a PRMT for Arg146 methylation of S3 *in vitro*

**Figure 2:**
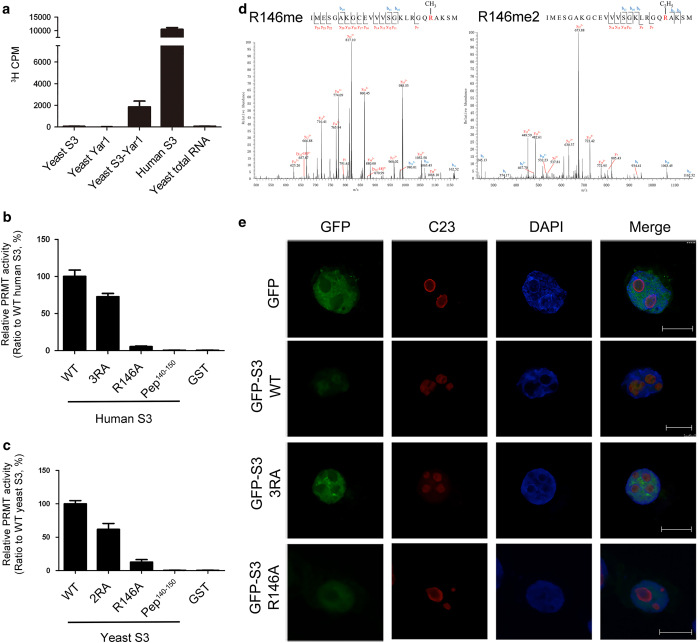
*In vitro* and *in vivo* functional assays of Sfm1 as a PRMT. (**a**) *In vitro* PRMT activity of Sfm1 towards yeast S3, Yar1 and the S3-Yar1 complex, human S3 and yeast total RNA. For all relevant panels in this figure, error bars are the s.d. from at least three replicates. (**b**) *In vitro* PRMT activity of Sfm1 towards the wild-type (WT), mutant (3RA and R146A) and a peptide (residues 140−150) of human S3. (**c**) *In vitro* PRMT activity of Sfm1 towards the WT, mutant (2RA and R146A) and a peptide (residues 140−150) of yeast S3. (**d**) LC−MS analysis of Arg146 methylation of human S3 catalyzed by Sfm1 *in vitro*. Left: monomethylation of Arg146; right: di-methylation of Arg146. (**e**) Subcellular locations of the WT and mutant (3RA and R146A) human S3. HEK293T cells are transfected with GFP, GFP-S3, GFP-S3 (3RA), and GFP-S3 (R146A). Cells are stained for GFP (green), C23 (red), and DAPI (blue). Bar: 10 μm.

So far, most of the SPOUT MTases are found to have activity only towards RNAs [8]. Intriguingly, our biochemical data show that Sfm1 could not catalyze methylation of total yeast RNA (Figure 2a), suggesting that it might not function as an RNA MTase. In the structure of Nep1 in complex with an RNA, the RNA substrate binds to a positively charged surface groove at the dimer interface and the dimerization is essential for substrate binding [29]. Electrostatic potential surface analysis of Sfm1 shows that the surface surrounding the active site is largely negatively charged, which is unsuitable for binding an RNA substrate (Figure 1c). In addition, superposition of the SAH-bound Sfm1 with the Nep1-RNA complex indicates that the CTD of Sfm1 has steric clashes with αA and αE of molecule B (corresponding to α1 and α6 of Sfm1) in the dimeric Nep1, which form the core of the dimer interface (Figure 1d). These results may explain why Sfm1 cannot form a similar homodimer as Nep1 and has no activity towards RNA.

Previously, two groups reported that Sfm1 might function as a PRMT: one group showed that Sfm1 could catalyze ω-monomethylation of Arg146 of yeast ribosomal protein S3 [21], and the other group found that Sfm1 could catalyze methylations of two proteins of about 20 and 30 kDa in the yeast extract *in vitro*, the latter of which has a similar molecular weight to S3 [34]. In order to examine the PRMT activity of Sfm1 towards yeast ribosomal protein S3, we attempted to purify recombinant yeast S3. Unfortunately, yeast S3 exists mainly as inclusion body; thus, we could not obtain large quantity of soluble yeast S3 and therefore could not assess the activity of Sfm1 on yeast S3 (Figure 2a). Previous studies showed that yeast Yar1 can function as a chaperone for S3 and prevent S3 from aggregation [35]. Thus, we co-expressed yeast S3 and Yar1 and were able to purify a small amount of the S3-Yar1 complex. The biochemical data show that Sfm1 displays a moderate activity towards the S3-Yar1 complex, but no activity towards Yar1 (Figure 2a). As human S3 could be purified to high quality and quantity, we also tested the PRMT activity of Sfm1 towards human S3. Interestingly, Sfm1 exhibits a high activity towards human S3 (about fivefold higher than that for the yeast S3-Yar1 complex; Figure 2a). Furthermore, our biochemical data show that the C-terminal truncation, different fusion tags and tag locations have no effects on its PRMT activity towards human S3 (Supplementary Figure S4d). As Sfm1 exists as a monomer in solution, these results also indicate that Sfm1 functions as a monomer *in vitro*.

Since Sfm1 has a high activity towards human S3, we used human S3 instead of the yeast S3-Yar1 complex as a surrogate for the yeast S3 substrate in the PRMT activity assay of Sfm1. Human S3 is found to have methylation modifications on Arg64, Arg65 and Arg67 in the KH domain (Supplementary Figure S5a), which could be catalyzed by human PRMT1 both *in vitro* and *in vivo* [25]. Arg64 and Arg65 of human S3 are strictly conserved in eukaryotes, whereas Arg67 is replaced with Asn67 in yeast S3; Arg146 of yeast S3 is also strictly conserved in other eukaryotes (Supplementary Figure S5b). To investigate whether Sfm1 can methylate a specific site or multiple sites of S3, we made human S3 mutants and detected their methylation levels by Sfm1. Compared with the wild-type (WT) protein, the R64A/R65A/R67A (3RA) mutant retains about 70% methylation level by Sfm1, whereas the R146A mutant has only background methylation level (Figure 2b). Similarly, compared with the WT yeast S3-Yar1 complex, the R64A/R65A (2RA) mutant complex retains about 65% methylation level by Sfm1, whereas the yeast R146A mutant complex has about 10% methylation level (Figure 2c). These results indicate that Sfm1 can methylate Arg146 of S3 as the major site *in vitro*. In addition, we tested the PRMT activity of Sfm1 towards both human and yeast S3 peptides (residues 140−150). The results show that only background level of methylation was detected, indicating that Sfm1 has no detectable activity towards these peptides (Figure 2b and c). These results suggest that Sfm1 might recognize Arg146 of S3 based on the tertiary structure rather than the sequence.

To verify the methylation of human S3 by Sfm1 *in vitro*, we subjected the S3 samples treated with or without Sfm1 to liquid chromatography–mass spectrometry (LC−MS) analysis. Our results confirm that Arg146 of S3 is the major methylation site by Sfm1 and further show that Arg146 can be both mono- and di-methylated (Figure 2d). Without treatment of Sfm1, no methylation of Arg146 was detected. However, in both cases, no methylation of Arg64, Arg65 or Arg67 was detected (data not shown). These results demonstrate that Sfm1 can specifically catalyze both mono- and di-methylation of Arg146 of human S3 *in vitro*.

### Sfm1 can form a complex with S3 *in vitro* and *in vivo*

To examine whether Sfm1 has direct interaction with human S3, we performed analytical gel filtration chromatography with purified recombinant Sfm1 and human S3. Our results show that Sfm1 exists as a monomer in solution with an apparent molecular weight of 28 kDa, and human S3 exists as a dimer with an apparent molecular weight of 60 kDa, which is consistent with the previous report [36]. The mixture of Sfm1 and human S3 (1:1 molar ratio) exhibits a single peak with an apparent molecular weight of 65 kDa, which contains both Sfm1 and S3 with a molar ratio of 1:1 as shown by SDS–polyacrylamide gel electrophoresis analysis, indicating that Sfm1 and S3 form a stable complex (Supplementary Figure S6a and b). These results also imply that formation of the Sfm1-S3 complex somehow prevents formation of the S3 homodimer, suggesting that the dimer interface of S3 is likely involved in interaction with Sfm1. Furthermore, our LC−MS analysis shows that Yar1 and Sfm1 are among the most abundant proteins co-purified with the GST-tagged S3 in yeast (Supplementary Table S2), which is also in agreement with the previous data showing that Yar1 and S3 co-exist in a complex [35, 37]. These results together demonstrate that Sfm1 and S3 can form a complex both *in vitro* and *in vivo*, which is likely biologically relevant.

### Mutation of Arg146 leads to a mainly nucleoplasmic localization of human S3

It was reported previously that a triple mutation of Arg64, Arg65 and Arg67 (3RA) of human S3 led to deficiency in its import into the nucleolus and failure of the ribosome assembly [25]. Our biochemical data show that Sfm1 can specifically methylate Arg146 of human S3 (Figure 2a and d). To investigate whether Arg146 methylation of human S3 is of any biological relevance *in vivo*, we expressed human S3 in HEK293T cells and analyzed the subcellular locations of the WT and mutant (3RA and R146A) S3 proteins. As expected, GFP-WT is mostly localized in the nucleolus (about 82.9%), and GFP-3RA is largely localized in the nucleoplasm but a small portion in the nucleolus (about 12.8%), consistent with the previous results [25]. Interestingly, GFP-R146A is also largely localized in the nucleoplasm and fails to be imported into the nucleolus (about 6.7%; Figure 2e). This mislocalization of human S3 indicates that Arg146 methylation might also have an important role in the import of human S3 into the nucleolus. The importance of Arg146 methylation of S3 is also supported by the observation that mutation R146A of yeast S3 affects the yeast growth [21]. These *in vitro* and *in vivo* functional data together suggest that Arg146 methylation of S3 is biologically relevant and may have an important role in its import into the nucleolus and thus in the assembly of the ribosome small subunit from yeast to human.

To verify whether yeast S3 can be methylated *in vivo*, we overexpressed yeast S3 and Sfm1 in YPH499 strain and analyzed the methylation sites of the crudely purified S3 using LC−MS. Unfortunately, we were unable to detect the peptide containing Arg146 probably due to abundant arginine and lysine residues in this region that are prone to proteolysis. However, our results indicate that Arg64 and Arg65 of yeast S3 are not methylated (data not shown), consistent with our biochemical data showing that Arg64, Arg65 and Arg67 of human S3 and Arg64 and Arg65 of yeast S3 cannot be methylated by Sfm1 *in vitro* (Figure 2).

### The active site of Sfm1 is very similar to that of PRMTs

The PRMTs identified so far all belong to the seven-beta-strand MTases and contain a conserved catalytic domain and a β-barrel domain to facilitate substrate binding [9]. Sfm1 is the first SPOUT protein that can catalyze protein arginine methylation. How Sfm1 binds the protein substrate and catalyzes the arginine methylation are yet unknown. To explore the substrate binding and catalytic mechanism of Sfm1, we compared the active site of Sfm1 with that of representative PRMTs (*Rattus norvegicus* PRMT3, *Homo sapiens* PRMT5 and *Trypanosoma brucei* PRMT7), SPOUT RNA MTase (*Saccharomyces cerevisiae* Nep1) and seven-beta-strand RNA MTase (*Methanocaldococcus jannaschii* Trm5).

**Figure 3:**
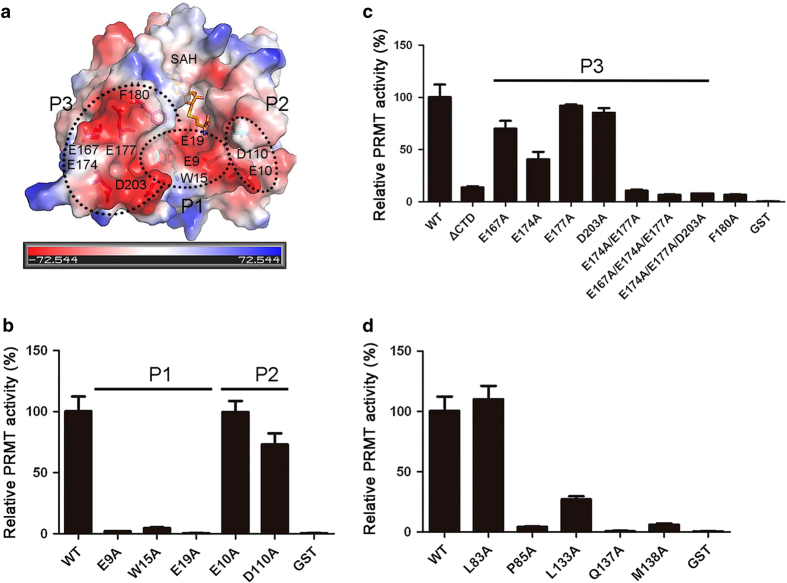
Analysis of the functional roles of the residues at the substrate-binding site of Sfm1. (**a**) Sfm1 exhibits a largely negatively charged surface surrounding the active site. The electrostatic potential surface is displayed as blue for positive, red for negative and white for neutral. The structure of Sfm1 is shown with a ribbon model and SAH is shown with a stick model. The acidic residues composing the potential substrate-binding site are divided into three regions (P1, P2 and P3) and are shown with stick models. (**b**) *In vitro* PRMT activity of the wild-type (WT) and mutant Sfm1 containing mutations of the residues in regions P1 and P2. For all relevant panels in this figure, error bars are the SD from at least three replicates. (**c**) *In vitro* PRMT activity of the WT and mutant Sfm1 containing mutations of the residues in region P3. (**d**) *In vitro* PRMT activity of the WT and mutant Sfm1 containing mutations of the residues at the cofactor-binding site.

Structural analysis shows that Sfm1 exhibits a negatively charged surface surrounding the active site (Figure 3a), which is similar to PRMTs (PRMT3, PRMT5 and PRMT7) [18, 20, 38] but different from RNA MTases (Nep1 and Trm5) that have a positively charged surface surrounding the active site suitable for RNA binding [29, 39]. The negatively charged surface of Sfm1, which is presumably the binding site for the substrate, is consisted of a number of acidic residues (Figure 3a). To examine the functional roles of these acidic residues, we divided them into three regions (P1, P2 and P3) according to their locations and performed mutagenesis studies to analyze their effects on the PRMT activity (Figure 3).

The P1 region comprises of residues Glu9 and Glu19 at the active site. Mutations E9A and E19A of Sfm1 completely abolish the PRMT activity, indicating that these two residues have critical roles in the substrate binding and/or catalysis (Figure 3b). The P2 region comprises of residues Glu10 and Asp110 of the SPOUT domain, which are located on the right side of the active site. Mutation E10A has no effect on the activity and mutation D110A has little effect on the activity, suggesting that these two residues have minor roles in the substrate binding and/or catalysis. The P3 region comprises of residues Glu167, Glu174, Glu177 and Asp203 of the CTD, which are located on the left side of the active site (Figure 3a). Deletion of the CTD (Sfm1ΔCTD: residues 1−154) disrupts its binding with S3 (Supplementary Figure S6c) and completely abolishes the PRMT activity towards S3 (Figure 3c). Consistently, although single mutations E167A and E174A have moderate effects on the activity and single mutations E177A and D203A have little effects on the activity, double mutation (E174A/E177A) or triple mutations (E167A/E174A/E177A and E174A/E177A/D203A) almost abolish the PRMT activity (Figure 3c). These results indicate that the acidic residues in the P1 and P3 regions are likely involved in the substrate binding and/or catalysis.

**Figure 4:**
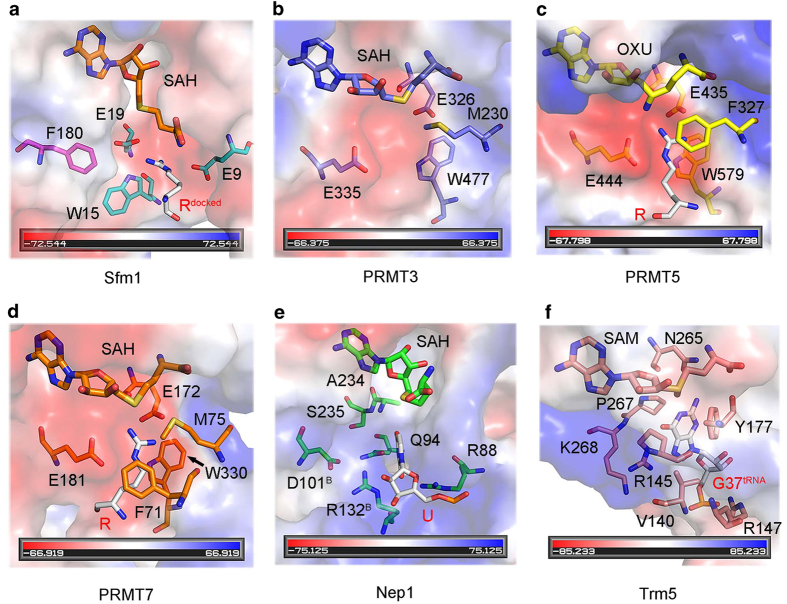
Structure of the active site of Sfm1 is similar to that of PRMTs. Structural comparison of the active site of Sfm1 with these of representative PRMTs and RNA MTases: (**a**) Sfm1. (**b**) PRMT3 (PDB identification (PDB ID): 1F3L). (**c**) PRMT5 (PDB ID: 4GQB). (**d**) PRMT7 (PDB ID: 4M38). (**e**) Nep1 (PDB ID: 3OIJ). (**f**) Trm5 (PDB ID: 2ZZM). The structures of Sfm1 and Nep1 (both belong to SPOUT MTases) are aligned using the secondary structure matching module of COOT [43], and the structures of PRMT3, PRMT5, PRMT7 and Trm5 (all belong to seven-beta-strand MTases) are aligned using the same method. The active site of each protein is shown with the electrostatic potential surface and the key residues, the cofactor SAM (S-adenosyl-methionine or its analog) and the substrate Arg are shown with stick models. The electrostatic potential surface is displayed as blue for positive, red for negative and white for neutral. The substrate Arg is docked to the SAH-bound Sfm1 structure using the HADDOCK server [46].

A detailed structural comparison shows that the active site of Sfm1 is very similar to that of PRMTs (PRMT3, PRMT5 and PRMT7) but substantially different from that of RNA MTases (Nep1 and Trm5; Figure 4). Previous structural studies have shown that the double E loop, the THW loop and the αY helix of PRMTs constitute the Arg-binding pocket adjacent to the cofactor-binding site and have important roles in the catalytic reaction [18, 20, 38]. In particular, the two strictly conserved Glu residues of the double E loop make hydrogen-bonding interactions with the guanidino nitrogens of the substrate Arg and the strictly conserved Trp residue of the THW loop makes hydrophobic interaction with the side chain of the substrate Arg. At the active site of Sfm1, there are two Glu residues (Glu9 and Glu19) that occupy similar spatial positions as the two Glu residues of the double E loop in PRMTs, and a Trp residue (Trp15) that occupies a similar spatial position as the Trp residue of the THW loop in PRMTs (Figure 4a and d). Although Sfm1 does not have a structure element equivalent to the αY helix of PRMTs, it contains a Phe residue (Phe180), which occupies a similar spatial position as Phe71 of PRMT7 that has hydrophobic interaction with the side chain of the substrate Arg [20]. Interestingly, these residues in Sfm1 are structurally arranged in a configuration that has a ‘mirror’ symmetry relationship with those in PRMTs (Figure 4a-d). A modeling study shows that the substrate Arg could be docked into the active site of Sfm1 very well (Figure 4a). Sequence alignment shows that residues Trp15, Glu19 and Phe180 of Sfm1 are all strictly conserved in different yeast species but Glu9 can be replaced with Asp in some species (Supplementary Figure S1b). The functional importance of these residues was examined by mutagenesis and *in vitro* PRMT activity assay. The results show that mutations E9A, W15A, E19A and F180A of Sfm1 completely abolish the PRMT activity, indicating that these residues have important roles in the substrate binding and/or catalysis (Figure 3b and c). Taking together, our structural and biochemical data suggest that Glu9, Glu19 and Trp15 compose the Arg-binding pocket and are directly involved in the recognition and binding of the substrate Arg and/or the catalysis, and that several acidic residues of the CTD on the surface are involved in the substrate binding and Phe180 of the CTD is involved in the binding of the substrate Arg.

In addition, we also verified the functional roles of the residues involved in the SAH binding (Figure 3d). Pro85, Leu133 and Met138 form a hydrophobic pocket to bind the adenine moiety of SAH via their side chains, and thus mutations P85A and M138A abolish the PRMT activity and mutation L133A retains about 25% of the activity. As Leu83 interacts with SAH via its main chain, mutation L83A has no effect on the activity. Although Gln137 has no direct interaction with SAH, it is located about 4.6 Å away from the sulfur atom of SAH and thus might be involved in the binding of SAM and/or catalysis, which is supported by the mutagenesis data that mutation Q137A completely disrupts the activity. These results are consistent with the previous structural and functional studies of other SPOUT MTases [27–30].

## Discussion

In this study, we report the crystal structure of yeast Sfm1, which consists of a typical SPOUT domain flanked by a small CTD. Our biochemical data show that the SPOUT domain-containing Sfm1 harbors PRMT activity towards both yeast and human S3 *in vitro* but has no activity towards yeast total RNA, which is in agreement with the previous report that Sfm1 is responsible for Arg146 methylation of yeast S3 [21]. In addition, our data show that Sfm1 can catalyze the mono- and di-methylation of Arg146 of human S3 *in vitro*, and Arg146 methylation might have an important role in the import of S3 into the nucleolus *in vivo*. These results clearly demonstrate that a SPOUT protein can function as a PRMT rather than an RNA MTase.

Most of the SPOUT MTases reported so far exist and function as homodimers and the dimerization is essential for the substrate binding and the activity [8]. Nevertheless, there is an exception: Trm10 and its homolog human TrmT10A (PDB identification (PDB ID): 4FMW) are both found to exist and function as a monomer [27]. In the Trm10 and human TrmT10A structures, there is an extra C-terminal α-helix (α6, existing only in the Trm10 family), which blocks the dimer interface in the dimeric SPOUT MTases. It is suggested that Trm10 might use a positively charged surface on the SPOUT domain to bind the tRNA substrate and the N-terminal extension might be involved in substrate binding [27]. Interestingly, Sfm1 contains an extra CTD, which also occupies the dimer interface in the dimeric SPOUT MTases, and thus exists and functions as a monomer as well. In addition, the CTD of Sfm1 forms part of the putative substrate-binding site and is critical for the PRMT activity (Figure 3c and Supplementary Figure S6c). So far, a large number of proteins were identified or predicted to contain SPOUT domain with unknown functions, many of which comprises additional motifs or regions at the N terminus or C terminus of the SPOUT domain and sometimes insertions in the SPOUT domain [8]. We expect that more SPOUT proteins will be found to function as PRMTs and to exist as monomers, in which the extra structure elements are likely involved in substrate binding (either RNA or protein). It is noteworthy that although Sfm1 acts as a PRMT and the active site of Sfm1 is similar to that of PRMTs, it exists and functions as a monomer. In contrast, all PRMTs reported so far exist and function as homodimers (or even oligomers) [9, 10]. Further structural and functional studies might be able to help understand the differences in the underlying molecular mechanisms of these enzymes.

In this study, we demonstrate that Sfm1 can specifically methylate Arg146 of both yeast and human S3 *in vitro*. Sequence alignment shows that Arg146 of S3 is strictly conserved in all eukaryotes (Supplementary Figure S5b). Intriguingly, all higher eukaryotes contain S3 orthologs but Sfm1 orthologs exist only in fungi (Supplementary Figure S1b). However, our *in vitro* and *in vivo* functional data indicate that Arg146 of human S3 could be methylated and Arg146 methylation has an important role in its import into the nucleolus, suggesting that higher eukaryotes might contain a specific or non-specific PRMT(s) responsible for Arg146 methylation of S3. To explore this possibility, we tested the PRMT activity of *R. norvegicus* PRMT1 (type I) and *H. sapiens* PRMT5 (type II) towards human S3. The results show that PRMT1 exhibits about 1/6 of the PRMT activity of Sfm1 towards S3, and PRMT5 about 1/10 of the PRMT activity of Sfm1 (Supplementary Figure S6d). In addition, both PRMT1 and PRMT5 show lower activities towards the 3RA and R146A mutants, suggesting that PRMT1 and PRMT5 could non-specifically methylate human S3 *in vitro* with low activity. Most recently, PRMT9 was shown to be another type II PRMT [11, 12], and S3 was found to be a possible interacting protein of PRMT9 based on MS analysis of the PRMT9 protein complex purified from Hela cells [11]. Further *in vitro* and *in vivo* functional studies are needed to find out whether PRMT9 can function as a PRMT for Arg146 methylation of human S3.

## Materials and Methods

### Cloning, expression and purification of proteins

The genes encoding the full-length yeast Sfm1 (residues 1−213), a C-terminal truncated Sfm1 (Sfm1ΔC, residues 1−204), the CTD deleted Sfm1 (Sfm1ΔCTD, residues 1−154), the full-length yeast Yar1 and the full-length yeast S3 were amplified by PCR from the cDNA library of *S. cerevisiae* and the gene encoding the full-length human S3 was amplified from the cDNA library of human HEK293T cells. Constructs of Sfm1 and human and yeast S3 mutants containing point mutations were generated using the QuikChange Site-Directed Mutagenesis kit (Strategene, La Jolla, CA, USA) and verified by sequencing.

For structural studies, the full-length Sfm1 and the Sfm1ΔC variant were cloned into a modified pSJ2 plasmid (Novagen, Madison, WI, USA) with a C-terminal His_6_ tag. The plasmids were transformed into *E. coli* BL21 (DE3) Codon-Plus strain (Novagen). The transformed cells were grown at 37 °C in LB medium containing 0.05 mg ml^–1^ ampicillin until OD_600_ reached 0.7, and the protein expression was induced with 0.25 mM IPTG at 16 °C for 24 h. The target proteins were purified by a combination of affinity chromatography using a Ni-NTA column (Qiagen, Hilden, Germany) and gel filtration chromatography using a Superdex 200 column (10/60; column volume, 120 ml; GE). Expression and purification of the Se-Met substituted Sfm1ΔC were the same as for the native protein except that the bacterial cells were grown in M9 medium containing amino acids Lys, Thr, Phe, Leu, Ile, Val, Se-Met and 1% lactose. The purified proteins were of >95% purity as evaluated by SDS–polyacrylamide gel electrophoresis and stored in the storage buffer (20 mM Tris-HCl, pH 8.0 and 300 mM NaCl).

For biochemical studies, the full-length Sfm1 and the Sfm1ΔCTD variant were cloned into the pGEX-6P1 plasmid (Novagen) with an N-terminal GST tag, and the human S3 was cloned into the pGEX-6P1 plasmid, the pSJ2 plasmid with an N-terminal His_6_ tag or pET-28a plasmid (Novagen) with a His_6_-sumo tag at the N terminus. Expressions of these proteins were performed as described above. The GST-tagged proteins were purified by glutathione sepharose beads (GE) and the His_6_-tagged proteins by Ni-NTA affinity chromatography. The His_6_-sumo tag at the N terminus of human S3 was removed by the Ulp1 protease. To prepare the yeast Yar1 and S3 complex, the yeast Yar1 and S3 were cloned into the pET-28a plasmid (Novagen) with a His_6_-sumo tag at the N terminus and the pGEX-6P1 with a GST tag at the N terminus, respectively. The two plasmids were co-transformed into *E. coli* BL21 (DE3) Codon-Plus strain (Novagen), and expressions of these proteins were performed as described above. The yeast His_6_-sumo-Yar1 and GST-S3 complex was purified by affinity chromatography using a Ni-NTA column (Qiagen). All the proteins were stored in the storage buffer.

For identification of possible proteins interacting with S3 in yeast, the yeast S3 and Sfm1 were cloned into a modified pYES2/CT plasmid (Invitrogen) with a GST tag at the N terminus and the pYES3/CT plasmid (Invitrogen) without tag, respectively. The two plasmids were co-transformed into yeast strain YPH499. The transformed cells were grown at 30 °C in the uracil and tryptophan selective medium until OD_600_ reached 0.7, and the protein expression was induced with 2% galactose at 30 °C for 12 h. The GST-S3 protein complex was purified by glutathione sepharose beads (GE) and analyzed by LC−MS.

### Crystallization, diffraction data collection and structure determination

Crystallization was performed using the hanging drop vapor diffusion method at 4 °C by mixing equal volumes (1.0 μl) of the protein solution (16 mg ml^–1^) and the reservoir solution. Crystals of the apo Se-Met and native Sfm1ΔC were obtained with the reservoir solution containing 0.2 M (NH_4_)_2_SO_4_, 0.1 M Bis-Tris (pH 5.5) and 25% (w/v) PEG 3350. Crystals of the SAH-bound Sfm1 were grown in drops consisting of the protein solution supplemented with SAH (1:3 molar ratio) and the reservoir solution containing 0.2 M sodium acetate, 0.1 M sodium cacodylate (pH 6.5) and 30% (w/v) PEG 8000. Diffraction data were collected from flash-cooled crystals at 100 K at BL19U1 of National Facility for Protein Science Shanghai and BL17U of Shanghai Synchrotron Radiation Facility, and processed using HKL2000 [40]. The statistics of the diffraction data are summarized in Table 1.

The apo Sfm1ΔC structure was solved by the single-wavelength anomalous dispersion method using Phenix [41]. The SAH-bound Sfm1 structure was solved by the molecular replacement method using the apo Sfm1ΔC structure as the search model. Structure refinement was carried out using Phenix [41] and Refmac5 [42]. Model building was performed using COOT [43]. Stereochemistry of the structure models was analyzed using Procheck [44]. Structural analyses were carried out using programs in CCP4 [45] and the PISA server [33]. Structure figures were generated using PyMOL (http://www.pymol.org). The statistics of the structure refinement and final structure models are summarized in Table 1.

### Analytical gel filtration analysis

Analytical gel filtration was performed using a Superdex 200 column (10/30; column volume, 24 ml) in AKTApurifier Chromatography FPLC system (GE). The buffer used for gel filtration consists of 20 mM Tris-HCl, pH 8.0, and 300 mM NaCl. In all of the experiments, 450 μl of protein sample were injected at a flow rate of 0.5 ml min^–1^. Standard protein samples were purchased from Sigma, St Louis, MO, USA and analyzed under the same conditions. The elution profiles were monitored by the absorption of ultraviolet at 280 nm.

### Dynamic light scattering analysis

Dynamic light scattering measurements were performed at the protein concentration of 4 mg ml^–1^ in 20 mM Tris-HCl, pH 8.0 and 300 mM NaCl, with DynaPro (Wyatt Technology, Santa Barbara, CA, USA). Each sample (Sfm1 or Sfm1 pre-incubated with SAH at 1:3 molar ratio) was measured 30 times and the results were analyzed with DYNAMICS V6 (Wyatt Technology, Santa Barbara, CA, USA).

### *In vitro* PRMT activity and GST pull-down assays

For *in vitro* PRMT activity assay, 1 μM of the WT or mutant Sfm1 or the WT Sfm1ΔCTD were incubated with 8 μM yeast S3, yeast S3-Yar1 or human S3 and 0.5 μCi [methyl-^3^H]-SAM (PerkinElmer, Akron, OH, USA) with a total volume of 40 μl in a reaction buffer containing 50 mM Tris-HCl (pH 8.0), 100 mM NaCl and 1 mM DTT at 30 °C for 2 h. Incubating samples on ice stopped the reaction. The reaction mixture was blotted on p81 paper. The free radio-labeled SAM was removed by washing three times using the buffer containing 100 mM NaHCO_3_ (pH 9.0). The PRMT activity was represented by the incorporated SAM as analyzed by liquid scintillation counting. The background reading of the assay system without adding the substrate was measured (around 200 c.p.m.) and subtracted from the reading of the activity assay. To examine the RNA MTase activity of Sfm1, the total yeast RNA was used as the substrate. All experiments were performed three times.

For *in vitro* GST pull-down assay, 20 μg GST-Sfm1 or GST-Sfm1ΔCTD were incubated with 100 μg human His_6_-S3 and 20 μl glutathione sepharose beads at 4 °C for 2 h. The beads were analyzed by SDS–polyacrylamide gel electrophoresis with Coomassie blue staining.

### Mass spectrometry analysis

For analysis of Arg methylation sites of human S3 catalyzed by Sfm1 *in vitro*, the protein samples were prepared as for the PRMT assay except that SAM was not radioactive labeled. The sample was digested with chymotrypsin (Roche, Basel, Switzerland) and analyzes by LC−MS with an EASY-nLC1000 liquid chromatography system and a Q Exactive mass spectrometer (ThermoFisher, Waltham, MA, USA). Peptides eluted from the liquid chromatography column were transferred directly into the mass spectrometer by electrospray with the application of a distal 1.8-kV spray voltage.

Protein identification and methylation analysis were performed with Integrated Proteomics Pipeline (IP2, http://www.integratedproteomics.com/) by searching against UniProt human database. Carbamidomethylation (+57.02146 Da) of cysteine was considered as a static modification, while mono- and di-methylation (+14.01565 and +28.0313) on arginine were considered as variable modifications. The database search results were assembled and filtered using the DTASelect program. All the methylation sites were firstly filtered using three parameters, the cross-correlation score (XCorr), the normalized difference in cross-correlation scores (DeltaCN) and the mass accuracy (p.p.m.). Peptides, which have the XCorr value higher than 2, the DeltaCN value higher than 0.1, and the mass accuracy lower than 15 p.p.m., were then checked manually to confirm the methylation sites.

For identification of interacting partners of yeast S3, the sample was digested with chymotrypsin and analyzed as described above. The results were searched against UniProt yeast database. Data were presented as a summary of top protein peptide hits identified.

### Confocal fluorescence microscopy analysis

GFP-tagged human S3 was cloned into the pEGFP-C3 vector (Clontech, Mountain View, CA, USA). HEK293T cells were cultured on coverslips in DMEM (Hyclone, Logan, UT, USA) supplemented with 10% fetal bovine serum (Biochrom, Berlin, Germany), pretreated with 10 mg ml^–1^ poly-D-Lys (Sigma) for 12 h, and then transiently transfected with the plasmid using lipofectamin 2000 (Invitrogen, Waltham, MA, USA). Thirty-six  hours after transfection, the cells were fixed with 4% paraformaldehyde at 25 °C for 20 min and washed three times with PBS. The cells were subsequently permeabilized and blocked with PBS-BT (1×PBS, 3% BSA and 0.1% Triton X-100) for 30 min at 25 °C. The coverslips were incubated with primary and secondary antibodies diluted in PBS-BT. Confocal images were obtained using a Leica TCS SP5 confocal microscope with a ×63 oil immersion lens. The localization markers for the nucleolus and nucleoplasm were C23 antibody and DAPI, respectively. Representative images from several independent experiments in which at least 100 cells were analyzed are shown as the results.

### Accession codes

The crystal structures of the apo Sfm1ΔC and the SAH-bound Sfm1 have been deposited with the Protein Data Bank under accession codes 5C74 and 5C77, respectively.

## Figures and Tables

**Table 1:** Summary of diffraction data and structure refinement statistics

	*Se-Met apo Sfm1ΔC*	*Native apo Sfm1ΔC*	*Native SAH-bound Sfm1*
PDB ID		5C74	5C77
			
*Diffraction data*			
Wavelength (Å)	0.9795	0.9792	0.9789
Space group	*P*4_1_2_1_2	*P*4_1_2_1_2	*P*2_1_
Cell parameters			
*a*, *b*, *c* (Å)	107.7, 107.7, 87.6	107.6, 107.6, 87.5	43.4, 95.6, 58.4
α, β, γ (Å)	90, 90, 90	90, 90, 90	90, 106.2, 90
Resolution (Å)	50.0−2.0 (2.1−2.0)^a^	50.0-1.9 (2.0-1.9)	50.0-2.5 (2.6-2.5)
Observed reflections	358 409	876 914	59 179
Unique reflections (*I*/σ(*I*)>0)	35 149	41 006	15 852
Average redundancy	10.2 (10.4)	21.4 (21.3)	3.7 (3.8)
Average *I*/σ(*I*)	20.9 (4.8)	38.3 (11.4)	14.9 (4.1)
Completeness (%)	99.3 (100.0)	99.8 (99.7)	99.6 (99.9)
*R*_merge_ (%)^b^	13.0 (47.9)	10.3 (32.0)	11.7 (38.9)
			
*Refinement and structure model*			
Reflections (*F*o≥0σ(*F*o))			
Working set		38 897	15 041
Test set		2 056	789
*R* factor/free *R* factor (%)^c^		19.3/23.2	21.1/26.7
Number of non-H atoms		3 393	3 426
Number of molecules/ASU		2	2
Number of amino-acid residues		211/144	203/199
Number of water molecules		443	77
Average B factor (Å^2^)			
All atoms		30.7	48.0
Protein atoms		29.4	46.8
SAH atoms			58.3
Water atoms		38.9	45.9
Root-mean-square deviations			
Bond lengths (Å)		0.007	0.006
Bond angles (°)		1.0	1.0
Ramachandran plot (%)			
Most favored regions		93.9	93.2
Allowed regions		6.1	6.8
Generously allowed regions		0	0

Abbreviations: PDB ID, PDB identification; SAH, *S*-adenosyl-homocysteine

^a^Numbers in parentheses represent the highest resolution shell.

^b^*R*_merge_=∑_hkl_∑_i_|I_i_(hkl)_i_−<I(hkl)>|/∑_hkl_∑_i_I_i_(hkl).

^c^*R*=∑_hkl_||F_o_|−|F_c_||/∑_hkl_|F_o_|.
