# Supplementary figures and images for: Structural basis for Sfm1 functioning as a protein arginine methyltransferase
Source: Cell Discov. 2015 Dec 29;1:15037–. doi: 10.1038/celldisc.2015.37 (PMC4860837; doi:10.1038/celldisc.2015.37)

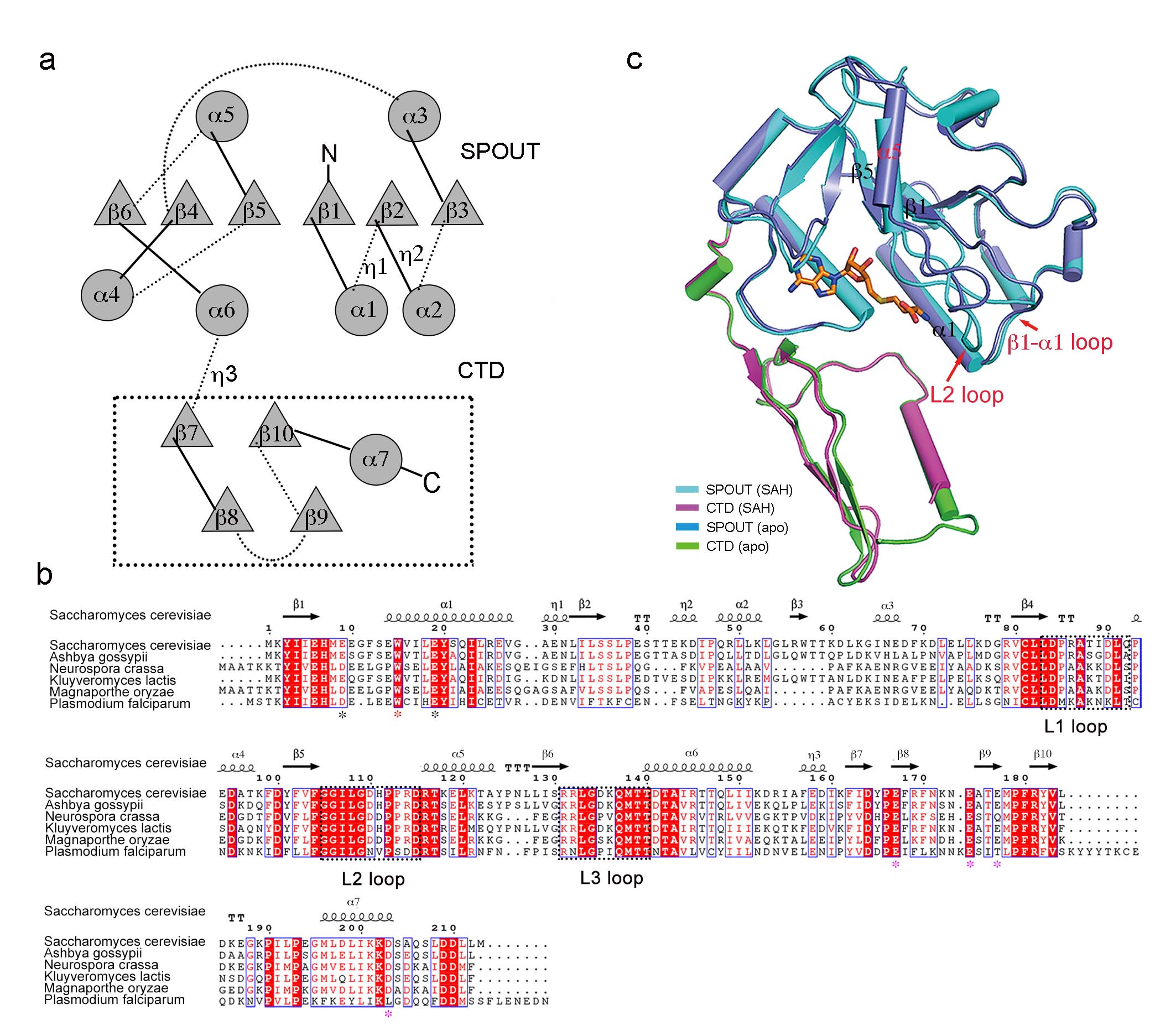

Supplement: Supplementary Figure S1 [file celldisc201537-s1.jpg]

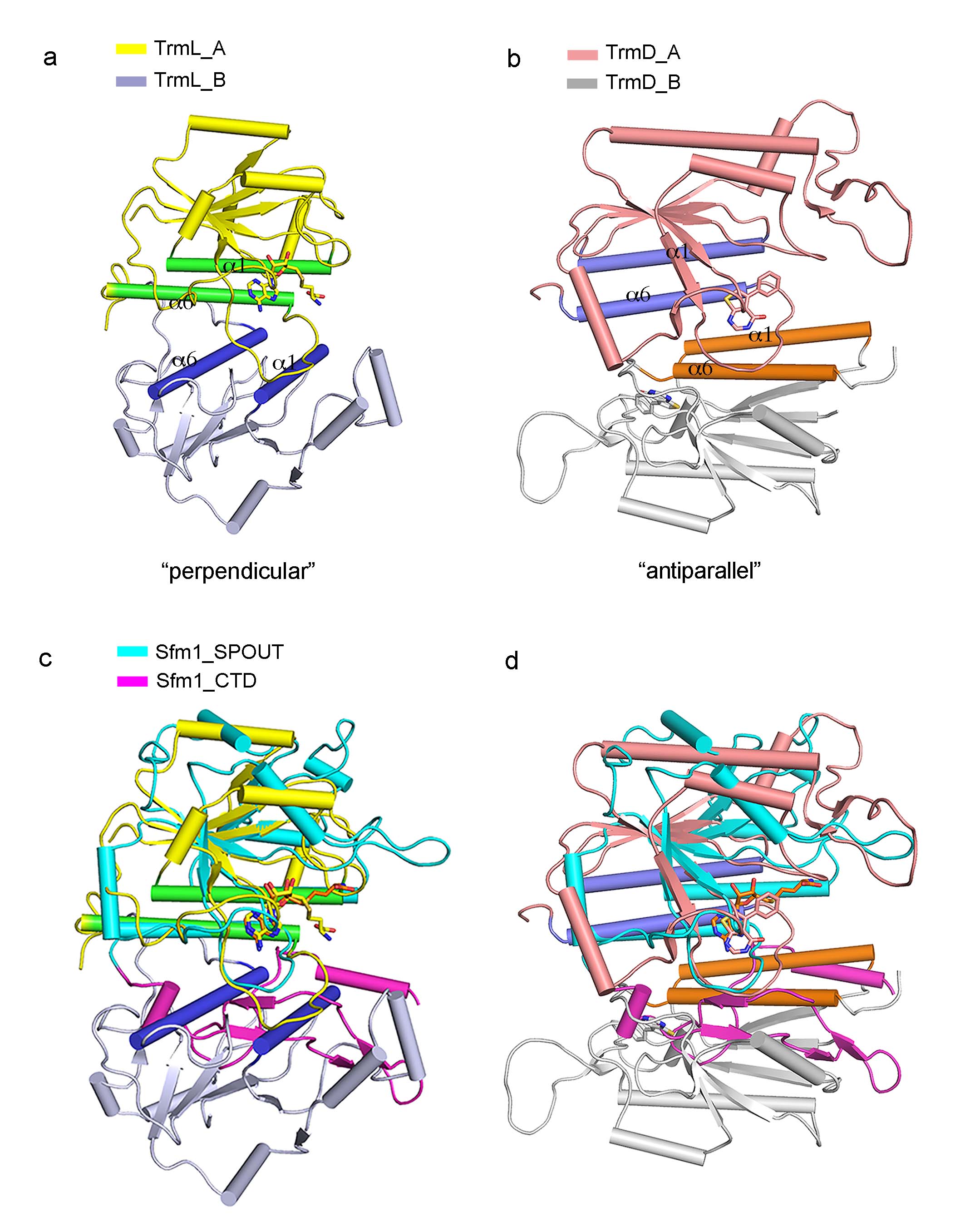

Supplement: Supplementary Figure S2 [file celldisc201537-s2.jpg]

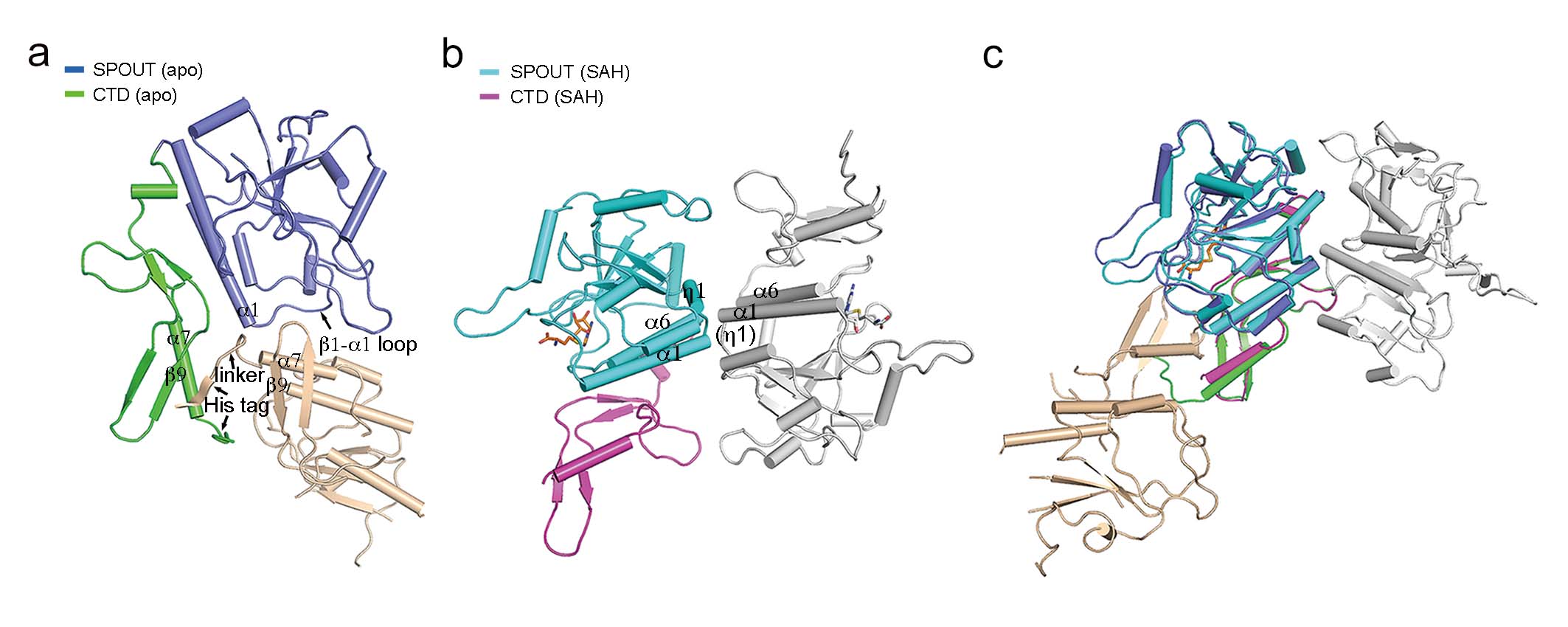

Supplement: Supplementary Figure S3 [file celldisc201537-s3.jpg]

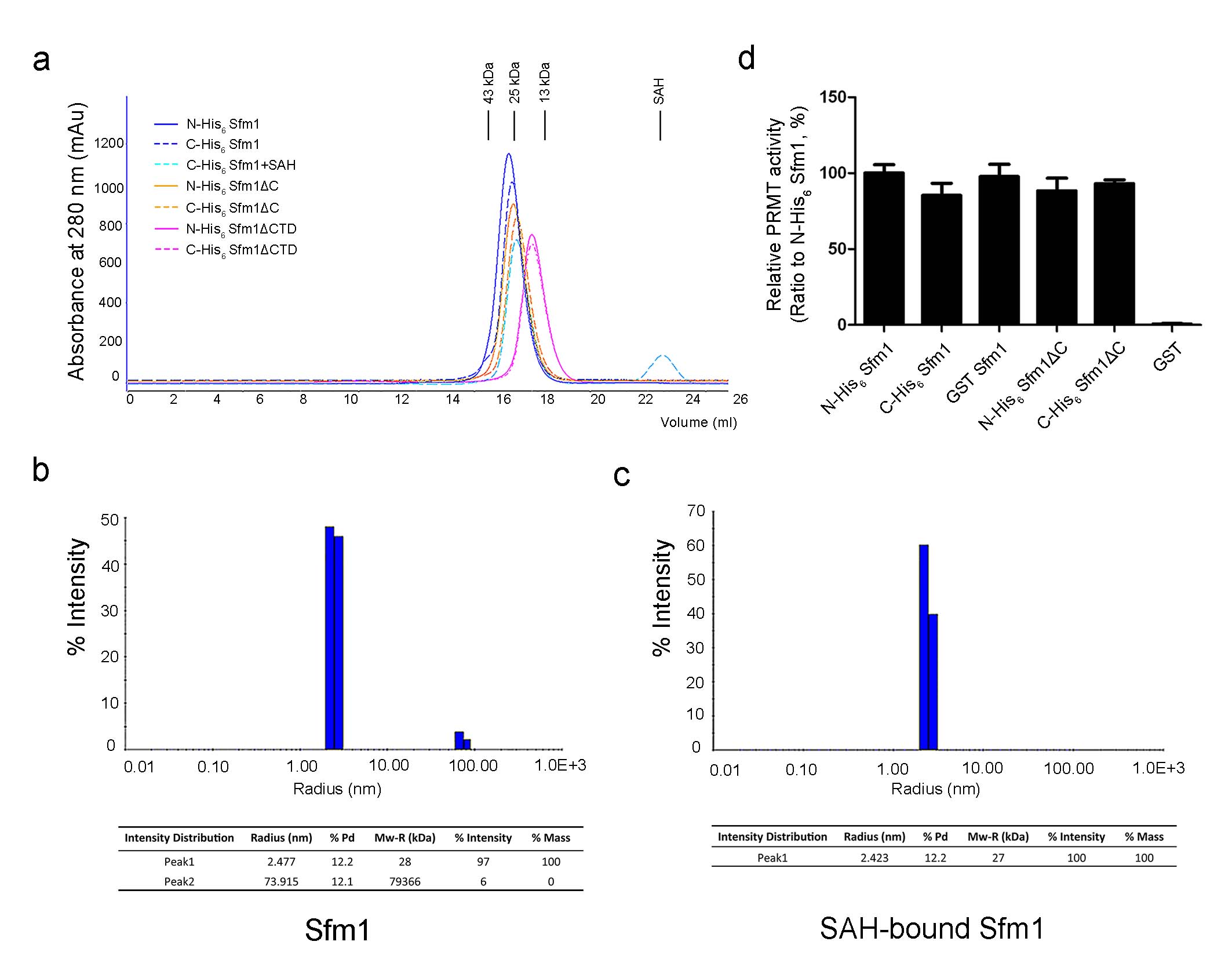

Supplement: Supplementary Figure S4 [file celldisc201537-s4.jpg]

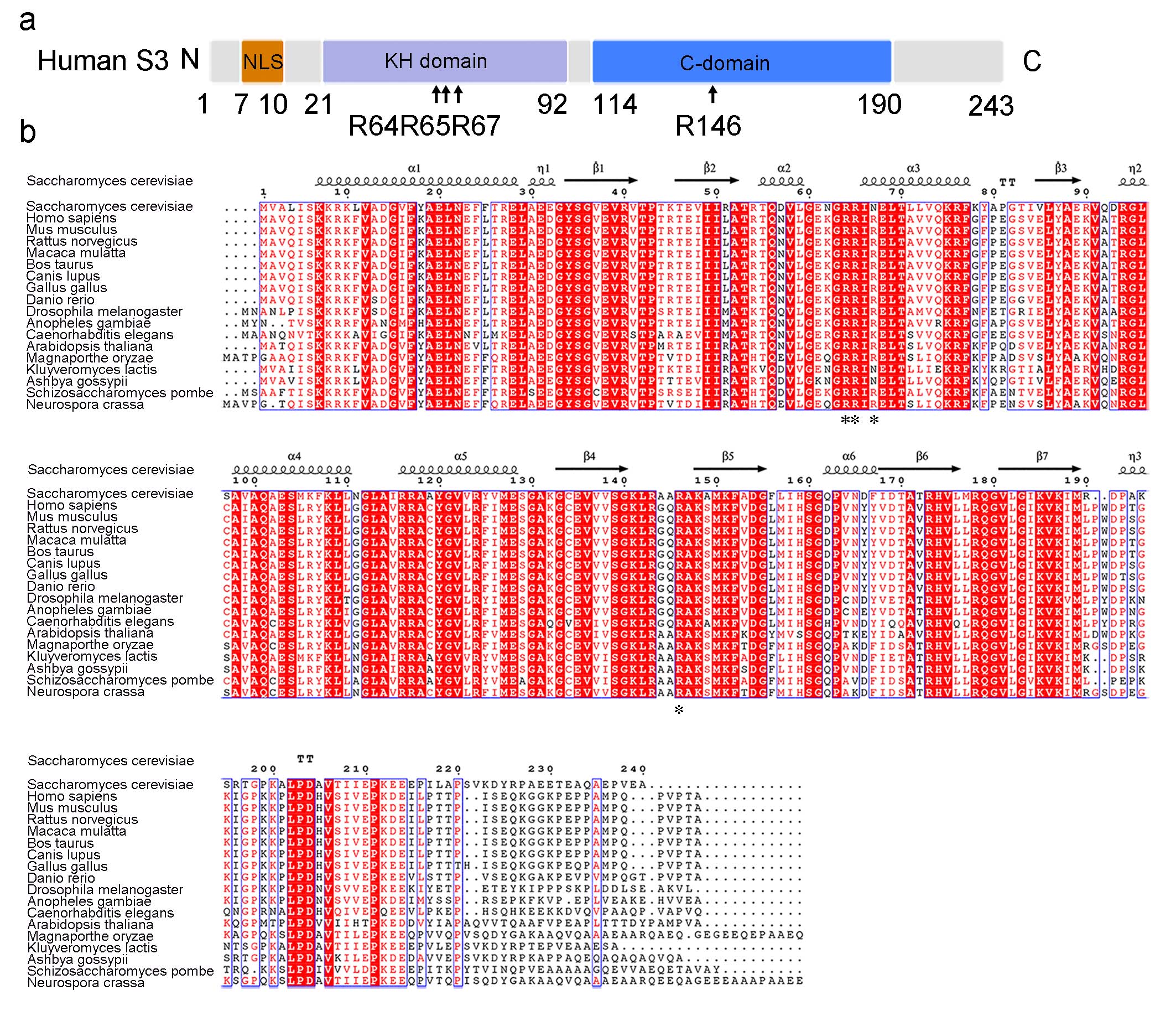

Supplement: Supplementary Figure S5 [file celldisc201537-s5.jpg]

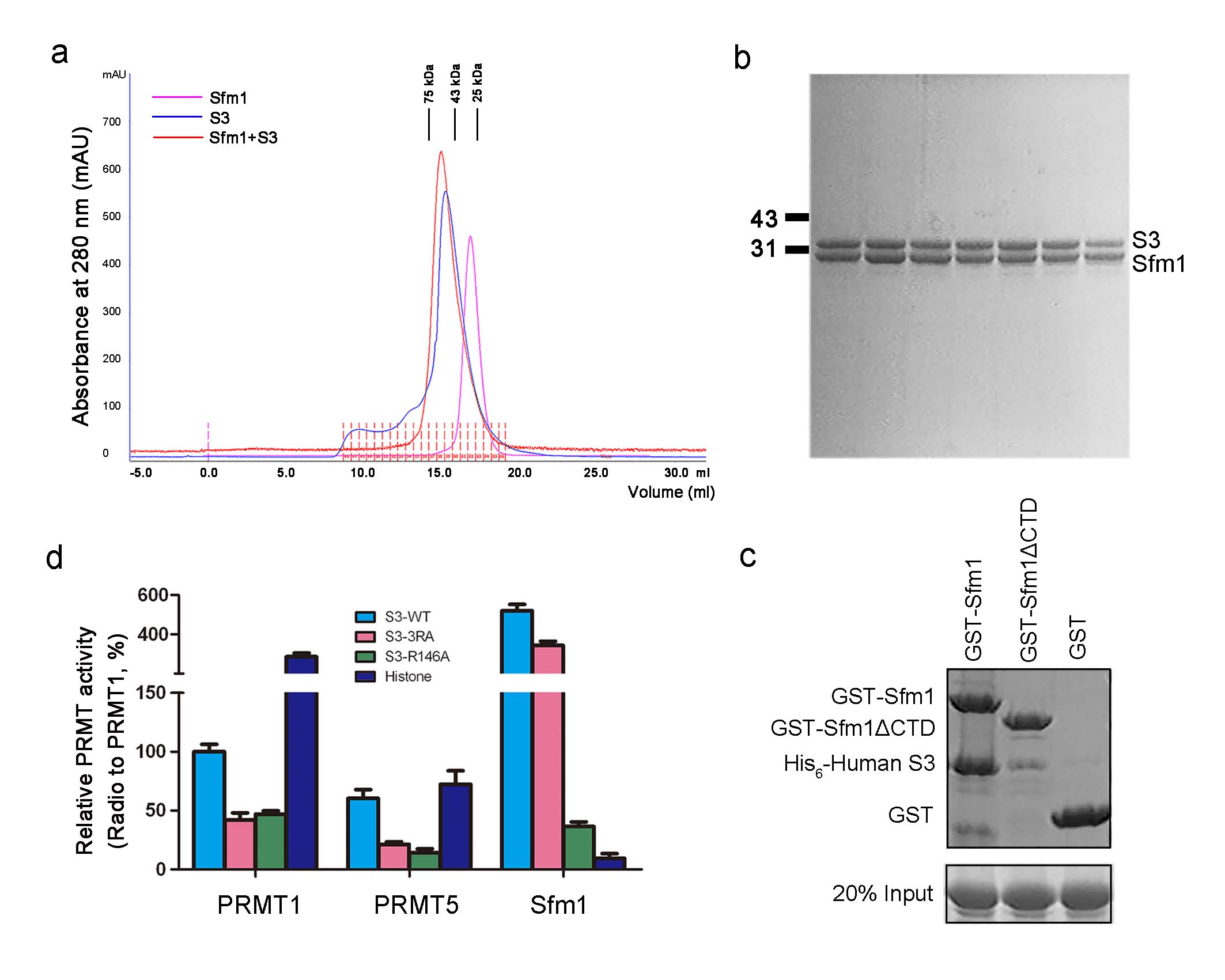

Supplement: Supplementary Figure S6 [file celldisc201537-s6.jpg]
