# Supplementary material for: Structural basis for Sfm1 functioning as a protein arginine methyltransferase
Source: Cell Discov. 2015 Dec 29;1:15037–. doi: 10.1038/celldisc.2015.37 (PMC4860837; doi:10.1038/celldisc.2015.37)
Supplement: Supplementary Table S1 [file celldisc201537-s7.pdf]

Table S1. Structural similarity of Sfm1 with representative SPOUT MTases

Protein	PDB ID	Z score	Rmsd (Å)	Nali	Nres	Identity (%)
Trm10	4JWF	10.2	3.1	137	188	9
TrmL	4JAL	9.6	2.8	120	156	17
Nep1	3OIJ	6.1	3.2	117	212	12
TrmD	4MCD	4.9	6.8	104	237	13
