# Supplementary material for: Structural basis for Sfm1 functioning as a protein arginine methyltransferase
Source: Cell Discov. 2015 Dec 29;1:15037–. doi: 10.1038/celldisc.2015.37 (PMC4860837; doi:10.1038/celldisc.2015.37)
Supplement: Supplementary Table S2 [file celldisc201537-s8.pdf]

Table S2. Identification of interacting partners of yeast ribosomal protein S3

Top hits (>4 identical peptides)	UniProt#	Mol Wt (Da)	No. identical peptides
S3	P05750	26503	11
Yar1	P46683	22356	8
GAPDH-3	P00359	35747	8
SSA1	P10591	69657	6
Sfm1	Q12314	24743	4
