# Supplementary material for: Structural basis for Sfm1 functioning as a protein arginine methyltransferase
Source: Cell Discov. 2015 Dec 29;1:15037–. doi: 10.1038/celldisc.2015.37 (PMC4860837; doi:10.1038/celldisc.2015.37)
Supplement: Supplementary Information [file celldisc201537-s9.pdf]

Supplementary Materials

Supplementary Figure S1 Structure of Sfm1. (**a**) Secondary structure topology of Sfm1. α-helices are shown as circles and β-strands as triangles. (**b**) Sequence alignment of Sfm1 with these in other fungi species. The sequences used in the alignment are: *Saccharomyces cerevisiae* (Sfm1, NCBI accession: NP_014664.1), *Ashbya gossypii* (NCBI accession: NP_982651.1), *Neurospora crassa* (NCBI accession: XP_960963.1), *Kluyveromyces lactis* (NCBI accession: XP_454372.1), *Magnaporthe oryzae* (NCBI accession: XP_368470.1) and *Plasmodium falciparum* (NCBI accession: XP_001350056.1). The alignment is performed using ClustalX [1] and displayed using ESPript [2]. The secondary structure elements of Sfm1 are placed on the top of the alignment. Strictly conserved residues are highlighted in shaded red boxes and conserved residues in open red boxes. The L1, L2 and L3 loops are highlighted by dotted boxes. The two Glu residues and the Trp residue at the active site are indicated by black and red asterisks, respectively, and the acidic residues of the CTD composing the potential substrate-binding site are indicated by magenta asterisks. (**c**) Superposition of the apo and SAH-bound Sfm1 structures. The structures of both apo and SAH-bound Sfm1 are shown with ribbon models and SAH is shown with a stick model. The SPOUT domain and the CTD of the apo and SAH-bound Sfm1 structures are colored in cyan, magenta, slate and green, respectively. The two structures can be superimposed very well with an RMSD of 1.2 Å for 200

Cα atoms. Structure elements with notable conformational changes upon SAH binding are indicated in red.

Supplementary Figure S2 Sfm1 exists as a monomer in the crystal structure. (**a**) Structure of dimeric TrmL (PDB ID: 4JAL) with the "perpendicular" manner. Monomers A and B are colored in yellow and blue-white, respectively. Two helices (α1 and α6) at the dimer interface are colored in green and blue, respectively. (**b**) Structure of dimeric TrmD (PDB ID: 4MCD) with the "antiparallel" manner. Monomers A and B are colored in salmon and grey, respectively. Two helices (α1 and α6) at the dimer interface are colored in slate and orange, respectively. (**c**) Superposition of Sfm1 and dimeric TrmL structures. (**d**) Superposition of Sfm1 and dimeric TrmD structures. The SPOUT domain of Sfm1 can be superimposed onto that of TrmL with an RMSD of 2.8 Å for 120 Cα atoms, and that of TrmD with an RMSD of 6.8 Å for 104 Cα atoms. All the structures are shown with ribbon models and SAH (or its analog) with stick models.

Supplementary Figure S3 Comparison of the intermolecular interface in the apo and SAH-bound Sfm1 structures. (**a**) The intermolecular interface of two molecules in the ASU in the apo Sfm1 structure. (**b**) The intermolecular interface of two molecules in the ASU in the SAH-bound Sfm1 structure. Sfm1 is shown with a ribbon model and SAH with stick a model. The SPOUT domain and the CTD of the apo and SAH-bound Sfm1 are colored in cyan, magenta, slate and green, respectively, and the other molecules are colored in wheat and grey,

respectively. (**c**) Comparison of the apo and SAH-bound Sfm1 structures based on superposition of one Sfm1 molecule.

Supplementary Figure S4 Sfm1 exists and functions as a monomer in solution. (**a**) Size-exclusion chromatography analyses of Sfm1 with different fusion tag locations and the C-terminal truncations, and in the presence or absence of SAH. Sfm1: residues 1-213; Sfm1ΔC: residues 1-204 and Sfm1ΔCTD: residues 1-154. The molecular weight markers are indicated. (**b**) Dynamic light scattering analysis of the C-terminal his_6 tagged Sfm1 in the absence of SAH. (**c**) Dynamic light scattering analysis of the C-terminal his_6 tagged Sfm1 in the presence of SAH. (**d**) *In vitro* PRMT activity of Sfm1 with or without the C-terminal truncation and with different fusion tags and tag locations towards human S3 Sfm1. Error bars are the SD from at least three replicates.

Supplementary Figure S5 Sequence comparison of S3 from different species. (**a**) A schematic representation of human S3. Arrows indicate locations of Arg64, Arg65, Arg67 and Arg146. (**b**) Sequence alignment of S3 in different organisms. The sequences used in the alignment are: *Saccharomyces cerevisiae* (NCBI accession: NP_014221.3), *Homo sapiens* (NCBI accession: NP_000996.2), *Mus musculus* (NCBI accession: NP_036182.1), *Rattus norvegicus* (NCBI accession: NP_001009239.1), *Macaca mulatta* (NCBI accession: XP_001086020.1), *Bos taurus* (NCBI accession: NP_001029219.1), *Canis lupus* (NCBI accession: XP_534008.1), *Gallus gallus* (NCBI accession: NP_001026007.1), *Danio rerio* (NCBI accession: NP_957447.1), *Drosophila melanogaster* (NCBI accession:

NP_476632.1), *Anopheles gambiae* (NCBI accession: XP_321155.3), *Caenorhabditis elegans* (NCBI accession: NP_498349.1), *Arabidopsis thaliana* (NCBI accession: NP_198403.1), *Magnaporthe oryzae* (NCBI accession: XP_360299.2), *Kluyveromyces lactis* (NCBI accession: XP_453432.1), *Ashbya gossypii* (NCBI accession: NP_982809.1), *Schizosaccharomyces pombe* (NCBI accession: NP_596763.1) and *Neurospora crassa* (NCBI accession: XP_956174.2). The alignment is performed using ClustalX [1] and displayed using ESPript [2]. The secondary structure elements of *S. cerevisiae* S3 are placed on the top of the alignment. Strictly conserved residues are highlighted in shaded red boxes and conserved residues in open red boxes. Locations of Arg64, Arg65, Asn67 and Arg146 in *S. cerevisiae* S3 are indicated by asterisks.

Supplementary Figure S6 Sfm1 and human S3 can form a complex and Arg146 of human S3 could be methylated by PRMT1 and PRMT5 *in vitro*. (**a**) Size-exclusion chromatography analysis of the mixture of Sfm1 (with a C-terminal 6xHis tag) and human S3 (without any tag). The elution profiles of Sfm1, human S3 and the Sfm1-S3 complex are shown in magenta, blue and red, respectively. (**b**) SDS-PAGE analysis of the elute sample collected at the peak of the size-exclusion chromatography profile of the Sfm1-S3 complex. The gel was stained by Coomassie blue. (**c**) *In vitro* GST pull-down assays of GST-fused Sfm1 and Sfm1ΔCTD with human His$_6$-S3. (**d**) *In vitro* PRMT activity of PRMT1, PRMT5 and Sfm1 towards human wild-type (WT) S3, mutants (3RA and R146A) S3 and histone. Error bars are the SD from at least three replicates.

References

1 Larkin MA, Blackshields G, Brown NP *et al*. Clustal W and Clustal X version 2.0. *Bioinformatics* 2007; **23**: 2947-2948.

2 Robert X, Gouet P. Deciphering key features in protein structures with the new ENDscript server. *Nucleic Acids Res* 2014; **42**: W320-324.
